# The Hospital. Nursing Section

**Published:** 1903-10-10

**Authors:** 


### The Hospital.

### Nursing Section.

Contributions for this Section of "The Hospital" should be addressed to the Editor, "The Hospital" Nursing Section, 28 & 29 Southampton Street, Strand, London, W.C.

No. 889.—Vol. XXXV. *SATURDAY, OCTOBER* 10, 1903.

### Notes on News from the Nursing World.

### NURSES NEEDED IN EUROPEAN TURKEY.

An appeal for help is made by members of the European Turkey Mission of the American Board of Commissioners for Foreign Missions, who entreat the Governments of Great Britain and the United States to take active measures to induce the Red Cross Societies of England and America to bring relief to the stricken districts which are in a state of insurrection. A passage in the appeal is to the following effect:—"The thought of whole districts weltering in blood without a single doctor or trained nurse to relieve the suffering is too broadly revolting to be tolerated in the twentieth century." No doubt there are English trained nurses ready to go to the Balkans if there is any organisation prepared to send them and competent to protect them.

### QUEEN ALEXANDRA'S IMPERIAL MILITARY NURSING SERVICE.

We are informed by the matron-in-chief of Queen Alexandra's Imperial Military Nursing Service that Miss M. L. Rannie, one of the matrons, has been placed under orders for service in Malta; that another matron, Miss A. E. Tait, has embarked for Indian troopship duty; and that Miss S. E. Webb, R.R.C., also a matron, has joined at Chatham for duty. Miss H. M. McCurdy has joined as sister at Gosport; Miss J. M. Clay, in a similar capacity, at Portsmouth; Miss E. M. Monck-Mason, at the Cadets' Hospital, Royal Military Academy, Woolwich, and Miss H. L. Neale, at the Royal Victoria Hospital, Netley. Staff nurse Miss Ada Ruth Myring has resigned her appointment; Miss A. F. Byers has embarked for Indian troopship duty as staff nurse, and Miss E. L. C. Crowe and Miss L. E. Mackay have joined as staff nurses at the Connaught Hospital, Aldershot, and Alton respectively.

### LADY DUDLEY'S NURSING SCHEME.

At a meeting in the Court House, Roscommon, last week, it was decided to appoint a district nurse under Lady Dudley's nursing scheme. It was stated that the proposal had met with very generous support in the town and locality. Annual subscriptions to the amount of £60 a year had been collected, and Lady Dudley had promised to make up the £100 required to support the nurse. Amongst other donations one had been received from the Mother Superior of the Convent. The selection of the nurse was left in the hands of Lady Dudley's committee, but it was stipulated that she should be a Roman Catholic and an Irish woman.

### THE BENEFIT OF ENGLISH TRAINING.

The matron of Visalia Sanitarium is advertising in our columns this week for a nurse who wishes to take up private work in California. She tells us that the American doctors prefer an English trained nurse if they can get one because they give English nurses credit for more common sense and less independence with regard to their orders than their American sisters. This, she says, accounts for the fact that "so many English and Canadian nurses in the Wild West are put in responsible positions in the different hospitals." The following fact illustrates the independence of some of the American nurses. In a San Francisco hospital, the head nurse held a clinic for the other nurses, dressing and bandaging a patient, who had recently undergone an operation and was not out of danger, five times at a sitting without the doctor's permission. An American physician, commenting on this incident, said, "That would never have happened if the nurse had received English training."

### DISTRICT NURSES IN THE CANTON OF BERNE.

We learn that district nurses for country parishes are scarce in Switzerland. One reason for this is that it is often difficult to give them sufficient occupation owing to the fact that registered midwives are established in every district, and therefore that work does not devolve on them. The pay is also very much lower than in England. A district nurse in the Canton of Berne who has been recently started receives no regular salary, but is paid "by the job." The district is some eight miles across. She is remunerated at the magnificent rate of 1½d. per hour by day and 1d. per hour by night. Some of the work which in England is done by the district nurse is undertaken in Switzerland by voluntary helpers. Samariter Posten, or First Aid stations, exist in most villages, with a Red Cross over the door. A person thoroughly versed in First Aid is prepared, if necessary, to administer it free of charge to anyone in need. He is generally the bootmaker or shopkeeper of the village. The materials are supplied by the Red Cross Society, and the patient is expected to pay if possible.

### GUARDIANS ON THE MARRIAGE OF NURSES.

At a recent meeting of the Londonderry Board of Guardians one of the nurses, a married woman, resigned her position as assistant nurse in the workhouse infirmary, and one of the Guardians in alluding to the matter proposed a resolution that in future "Any nurse or other female official in the service of the Guardians who intends getting married must previously tender her resignation to the Board." The need for this resolution had, he said, been arrived at not because the Guardians wished to prevent their nurses from getting married, but because it did not tend to efficient work and discipline in the infirmary.

to have married nurses who were naturally visited by their husbands. Another member, in seconding the proposal, observed that he had been on a committee a few years ago to investigate some charges brought forward by a Guardian. He found that the only real cause for the interference of the Guardians was on account of the visitors to the nurses and other officials, and it was then contended that as the nurses had ample time for social duties and pleasure it was not for the good of the house that there should be indiscriminate visiting. The Guardians decided that "they had no objection to the nurses courting all they want to outside and when off duty, and finishing off by getting married; but they must do no courting in the house." The resolution was agreed to, but the suggestion that the courting is done by the nurses themselves is gratuitously unkind.

### THE NEW MATRON OF BETHNAL GREEN INFIRMARY.

The vacancy caused by the resignation of Miss Hopper, who has been matron of Bethnal Green Infirmary since the commodious and up-to-date new building was opened, has been filled by the appointment of Miss Elizabeth Dodds. During Miss Hopper's absence from illness Miss Dodds has undertaken the duties of acting matron and performed them so efficiently that the infirmary committee of the Bethnal Green Board of Guardians could not have done better than recommend her for the permanent position. The salary of the matron is £100 a year with the usual allowances.

### NURSING AND PHYSICAL STRENGTH.

The futility of endeavouring to become a trained nurse without an adequate measure of physical strength has recently been shown in the case of the daughter of the rector of a country parish near Morecambe. The young lady, who was 28 years of age, took a great interest in hospitals, and decided to become a nurse. She started work in one of Dr. Barnardo's Homes, but only remained a few weeks. However, a little later she went as probationer to the Guest Hospital at Dudley, and continued her duties there for seven months. But at the end of that time her health again failed, and she was obliged to return home. One morning in September she was missing from the house soon after breakfast, and a telegram to the anxious father disclosed the fact that she had gone back to the Guest Hospital, apparently hoping that she might again try to nurse. When she was told that she must first apprise her parents of her intention she seemed extremely disconcerted, and walked away dejectedly from the institution. She wandered twenty miles away from Dudley, and when found there three days later was wet to the skin, having slept on the roadside at night. The matron of the Dudley Hospital, as well as the relatives, had been indefatigable in trying to find her. Her father's explanation is that her disappointment had resulted for the time being in nerve trouble; but the incident emphasises the importance of the utmost care being taken to ascertain that applicants for admission as probationers are physically qualified for the work.

### A LADY DOCTOR AS SUPERINTENDENT NURSE.

The Lurgan Board of Guardians have recently chosen Dr. Adams, a lady doctor, as resident physician. It appears that she is also expected, we presume for the sake of economy, to discharge the duties of superintendent nurse. We are glad to see, however, that Miss Carleton, a member of the board, who elicited this information, has protested against the dual appointment. She contended that unless a woman has been trained as a nurse she cannot be a nurse and cannot train nurses, any more than an architect can do the work of a bricklayer although he is competent to judge of the quality of a bricklayer's work. The contention is unanswerable. However well qualified Dr. Adams may be to act as resident physician she cannot, unless she has learnt nursing in practice as well as in theory, be recognised as a nurse.

### A VICTORIAN NURSE IN FORT FRANCES.

Nursing in the less-populated parts of Canada is often hard work, but few nurses have the experiences which fall to the lot of the Victorian Order nurse who during the last four years has worked at Fort Frances, Ontario. Up to the beginning of the winter of 1901–2 the place was completely isolated for at least seven months of the year, as the nearest railway station was 160 miles away, to reach which a journey by stage, and over many miles of ice on river and lake was necessary. Since the Canadian Northern Railroad was completed there has been a train service three times a week. The whole of the work for the patient, as well as the nursing, devolves upon the nurse, for there are few homes where a grown-up daughter can be found, and there is no paid help obtainable. When possible the nurse stays continuously with her patient—the population of the town is about 500—but when much pressed she goes from house to house. During last winter a critical case had to be visited three times a day, and the river could only be crossed by ferry. The first visit was made at 6 A.M.; the river was then frozen over, and the boat had to break the ice as it went. At 2 P.M. all was straight sailing, but at 9 P.M. there was frequently a dense fog, with the ever present danger of going over the falls if the boat got out of its course.

### GUARDIANS AND QUEEN'S NURSES IN SHEFFIELD.

An influential deputation from the Sheffield Queen Victoria Jubilee District Nursing Association, waited upon the Ecclesall Bierlow Guardians last week in order to ask the latter to consider certain proposals concerning the district nursing of the sick poor within the Union. Mr. Alderman Franklin stated that it was the wish of the Association that the Guardians should co-operate with them in their endeavour to bring about a scheme whereby people who are in poor circumstances, yet not in receipt of relief, may be attended by the district nurse. He said that the cost of each district nurse was about £100, and that if the Guardians would subscribe £50, the Committee would find the additional £50 required. The probable result of the discussion which ensued, will be that the Board will subscribe £200 a year from December 1st, the Committee of the Association agreeing to take over the two district nurses now employed by the Board, if the latter are willing to become Queen's nurses.

### AN INCIDENT AT AN ISOLATION HOSPITAL.

It will be recollected that a fortnight ago we called attention to the fact that the Chairman of the Billericay Board of Guardians had condemned the nurses at the Isolation Hospital, because, owing to sufficient reasons, they would not receive a patient suffering from enteric fever and sent him to the Union Infirmary. The Chairman also put in the extraordinary plea that a good many persons in the workhouse were endangered by the reception of a patient suffering from enteric fever in the infirmary. In reference to this contention, Dr. Carter, the medical officer, desires that it should be known that the nurses were not only willing to have the case, but were anxious to have it, as, in fact, he ascertained before sending it to the infirmary. We did not for a moment suppose that the infirmary nurses shared the strange views of the chairman of the Board.

### THE WORKMEN'S NURSE AND HER BADGE.

The authorities of the Queen's Jubilee Nursing Institute have been asked to reconsider their decision respecting the claim of Miss Violet Wood to the badge which is awarded to a Queen's nurse who has served for six years, and we hope that they will see their way to accede to the application. The only reason why Miss Wood has not already received the badge appears to be because she severed her connection with the District Nursing Association at Bishop Auckland, owing to circumstances already stated in our columns, and accepted the post of nurse under the newly-formed Auckland and District Working Men's Association. It is a pity that there has been a split, but as Miss Wood has been a Queen's nurse in Bishop Auckland for more than eight years, during which period she has gained general confidence and the affection of the working men in the town, we think that her title to the possession of the badge is made out.

### NURSES AND SWIMMING.

The beginning of October brings the nurses of the Portsmouth Parish Infirmary to the commencement of their winter session and the conclusion of their swimming lessons. During the summer a class of about twenty has learned to swim, a swimming-bath belonging to the schools being put at their disposal for some hours on certain days in the week, with the head male attendant—a past master in the art—as teacher. The nurses have greatly enjoyed the privilege and have been most persevering in their efforts to master this new accomplishment.

### A NURSES' HOME FOR SMETHWICK.

The foundation-stone of a nurses' home in connection with the Smethwick District Nursing Association, the gift of the Mayor, has just been laid by the Mayoress. The building is to be on Bearwood Hill, will be of three storeys, and will contain 14 rooms. The Mayor has also generously offered to defray the cost of furnishing the home, and altogether about £2,000 will be expended on the undertaking. Dr. Marsh Jackson, who presided at the ceremony, paid a high tribute to the work which is being carried on in the borough by the district nurses.

### A FLOURISHING VILLAGE NURSING ASSOCIATION.

The second annual report of the Whiston Nursing Association has been sent to us, and we gather from the details given that the movement so far has proved very successful. The villagers become members of the Association by subscribing a shilling a quarter, and this entitles them to the services of the nurse gratuitously at any time they require her. Of course, their subscriptions have to be supplemented by outside contributions, but the net financial result of the second year's transactions is that the committee have a balance in hand of £30. As the nurse, who holds the L.O.S. certificate, and has by degrees obtained almost all the maternity work in the village, receives a salary of £66 a year with free furnished rooms, and half the maternity fees—which are 7s. a case—the position is satisfactory all round. Whiston sets an example worthy of imitation not only to villages in Yorkshire but to many in various other parts of England.

### FUNERAL OF A NURSE AT YORK.

The mortal remains of Sister Rose, of the Community of the Holy Cross, sister-in-charge of the York Home for Nurses, were laid to rest on Saturday week amid every manifestation of regret and respect. The mourners in York Minster included the Dean, several of the clergy and lay members of the Council of the home, most of the nursing staff, and many other nurses. The Dean himself read the lesson. Beautiful wreaths and crosses sent by the friends of the sister covered the coffin, which, after the service, was conveyed to York Cemetery, where a concourse of people representing all classes of society assembled.

### THE PENSION FUND'S BONUS ADDITIONS.

So many nurses have written to the secretary of the Royal National Pension Fund respecting their bonus additions that it has been impossible for Mr. Dick to communicate with each personally. He asks therefore to be permitted, through our columns, to thank them for the kind things they have said about the Fund and its management.

### THE "HANDY WOMAN" NOT EXTINCT.

The need which still exists for skilful midwives amongst the poor was forcibly illustrated the other day. The matron of a nursing home in Cheshire was asked to send a nurse for a maternity case. Upon her arrival the nurse found that the patient was suffering from puerperal fever, and the "handy" woman who had delivered the patient informed the nurse that she had been douching her twice daily with soap and water. The same remedy had been applied regularly to the baby's eyes, which were extremely bad with ophthalmia.

### PLAISTOW DISTRICT NURSES.

The annual bazaar in aid of the District Nurses' Home at Plaistow will be held in the Public Hall, Canning Town, on November 24th, 25th and 26th. Nurses who have been trained at this institution may be glad to know that gifts of articles for such will be gratefully acknowledged if sent to Sister Katherine at Plaistow.

### The Examination of the Urine.

By J. K. WATSON, M.D.

(*Continued from Vol. XXXIV., page 326.*)

PART III.

THE COLLECTION AND EXAMINATION OF THE URINE.

There are certain points to be observed in the obtaining of a specimen. As a rule, a specimen taken from the mixed urine of 24 hours is examined; but it may be necessary to examine a specimen of urine passed at a special time, for instance, first thing in the morning or after food. The fresh urine should be placed in a tall, clean, conical glass, covered over with a porcelain slab or a piece of clean white paper to keep out dust, and set aside in a cool place. Time is allowed for any sediment to settle to the bottom of the glass. There are a large number of chemicals used for the examination of the urine, and the same applies to the apparatus used. In the following list the more commonly used ones are enumerated. Many of these are supplied in the various urine test stands on the market.

At least half a dozen test tubes; a urinometer with a stem graduated to 1060 in a case; a cylindrical glass to hold at least two ounces for taking the specific gravity; one or more tall conical glasses in which to collect the urine; spirit lamp; red and blue litmus paper and yellow turmeric paper in books; methylated spirit; small glass stoppered bottles containing (and labelled): nitric acid, acetic acid, picric acic, liquor potassæ[1] (caustic soda); Fehling's solution "No. 1"; Fehling's solution "No. 2"; Pavy's solution (may be used *vice* Fehling's); sulphate of copper (1 per cent. solution); ferrocyanide of potassium (5 per cent. solution); ozonic ether; tincture of guaiacum; a pipette; a small glass funnel; a glass stirring rod; filter papers; blotting paper; test tube holder; Esbach's albuminometer, and solution for same; Doremus's ureometer, and solution for same.

I. The quantity passed in the 24 hours should, as a rule, be recorded, where this is possible.

II. Describe the colour.

III. Describe the appearance, for example, whether clear, thick, ropy, or frothy.

IV. Take the specific gravity (sp. gr.). Clean and dry the urinometer and the urine glass, and nearly fill the glass with the urine, removing any froth from the surface with blotting paper. Then gently lower the urinometer into the glass, taking care that it floats and does not touch the sides of the glass. Look through the fluid and read off the number on the stem of the urinometer at the level of the fluid. Confirm this result by pushing down the urinometer and allowing it to rise again, and, when it is steady, reading the number again. In cases where two ounces of urine are not available, add an equal quantity of water to the urine, or, if necessary, even three parts of water to one of urine. Take the specific gravity of the mixture and multiply the last two numbers by two or by four according as one or three parts of water have been added. Thus, if half an ounce of urine and an ounce and a half of water have been mixed the dilution is 1 in 4. Suppose the sp. gr. to be 1007, then the true sp. gr. would be 1028 ($07 \times 4 = 28$).

V. Test the reaction. Blue litmus paper is turned red (acid reaction) by acid urine, and red litmus paper is turned blue (alkaline reaction) by alkaline urine. If neither litmus paper is altered in colour the urine is neutral. Occasionally the same urine will present both the alkaline and the acid reaction (amphoteric reaction). This, which should be recorded, is, however, of no special importance. If red litmus paper which has been turned blue by alkaline urine be heated or even allowed to dry in the air and the red colour returns this shows that the alkalinity is due to ammonia; in such a case, however, we should probably be able to tell the presence of ammonia by smelling the urine. It should be noted whether the change produced in the litmus paper is slight or well-marked; for example, the urine may be slightly or markedly acid. Yellow turmeric paper is turned brown by alkaline urine.

VI. Odour. Make a note of this; whether the urine has a natural, a sweet, or an offensive odour, or any other characteristic in this respect.

VII. Deposit. Describe the character, the colour, and the amount of any deposit; for example, the brick-red deposit of urates, or the dirty greyish-white deposit of phosphates.

VIII. Next test for albumin. This should never be omitted, and in so doing we may be able to throw light on the nature of any deposit which may be present. There are certain fallacies connected with nearly every test which is employed in examining urine, and it is partly on this account that we employ more than one test so as to make assurance doubly sure. When albumin is present in considerable amount it is perfectly easy to detect it, but it is also important to be able to detect faint traces of it, and sometimes this is a difficult matter. When there is more albumin than a trace it is necessary to estimate its amount.

To ascertain its presence the urine must be filtered, unless it is quite clear. It may be necessary to repeat this, and if then the urine be still opaque, a little caustic potash or caustic soda should be added (liquor potassæ or liquor sodæ), which produces a precipitate (cloud) of phosphates, and this precipitate renders the urine above it clear, which can be pipetted off into a test tube as required. If the urine be alkaline, it should be rendered acid by adding a few drops of acetic acid, since albumin is not precipitated by heat if the urine is alkaline. Now boil an inch of urine in a test tube. If it remains quite clear there is no albumin. If it becomes turbid this is probably due to albumin. It may possibly be due to phosphates; add one drop of nitric acid, and if the precipitate still remains, then we know it must be due to albumin, and vice versâ.

To confirm this result we shall mention three other tests, the most important of which is that known as the cold nitric acid test, called after Heller, who described it.

Take about half an inch of nitric acid in a test tube, and slant the tube; while holding it in this position pour down the side of the tube very carefully a few drops of the urine. Where the urine joins the acid a whitish ring is produced if albumin is present. The urine should not be allowed to mix with the acid. A pipette may be used to drop the urine on to the acid. It nearly always happens that this ring forms at once, or within a minute, and this test is so delicate that even traces of albumin may be discovered. Acid urates, urea, or certain balsams (such as cubebs or copaiva, which have been taken in medicine) may produce with this test a white cloud, but there should seldom be any difficulty in detecting albumin, since heat will nearly always dispel such a cloud, whereas it has no effect on the ring produced by albumin.

The next test is known as the picric acid test. Place about two inches of the acid in a test tube and drop the urine drop by drop on to the acid with a pipette. If there is no cloud formed there is no albumin. If there is a cloud formed then apply heat, and if the cloud remains

[1] The bottle containing liquor potassæ should be green tinted.

### THE EXAMINATION OF THE URINE—*Continued.*

albumin is present, if it disperses the cloud is caused by other substances. Lastly, we may make use of the ferrocyanide of potassium test.

Take the same quantity of urine as in the previous test, and add about 10 drops of the ferrocyanide and then some acetic acid. Turbidity shows the presence of albumin. The urine must be strongly acid for this test to succeed. Dr. Pavy uses pellets of citric acid, and of the ferrocyanide, which are convenient. The citric acid pellet is first dropped in and allowed to dissolve. If a cloud now forms, this is not albumin but mucin or urates. In such a case start afresh, and if we are dealing with mucin, add the two pellets together to a fresh lot of urine, and compare the precipitate caused by the mucin and that produced by the albumin. If urates are thrown down by the citric acid pellet, add to a fresh supply of urine an equal amount of water, and then on adding the citric acid there will be no cloud. After the citric acid pellet has dissolved, add the ferrocyanide pellet, and then the cloud of albumin, if any be present, will appear.

To estimate the amount of albumin, we use Esbach's albuminometer, which is a thick test-tube half an inch in diameter and about eight inches long, having a mark U at a level of about $2\frac{1}{4}$ inches, and a mark R about $1\frac{1}{2}$ inches higher. The tube is filled with urine up to U, and the special solution (one part of picric acid and two parts of dried citric acid in 100 parts of water), called after Esbach, is added up to R (reagent). A rubber cork is now fitted into the mouth of the tube (or the tube may be closed with the finger), and the tube is gently moved up and down, but not shaken, to mix the two fluids.

It is then put aside for 24 hours to allow the albumin to settle. By this time we can read off the level of the albumin, as the tube is graduated from one up to 12, each space representing one part per thousand of urine. Supposing the albumin reached to the figure 5, we should say that there were 5 parts of albumin per thousand or 0·5 per cent. As there are 437·5 grains in an ounce we can estimate the grains of albumin passed in the 24 hours. If 50 ounces containing this amount of albumin per cent. were passed, then the grains would be $0·5 \times 4·375 \times 50 = 109·375$.

Esbach's albuminometer will not estimate traces of albumin (the least amount recorded is 0·1 per cent.). Remember to filter the urine here, as before, if it is not clear, and also to render it acid with acetic acid if it be alkaline. If the sp. gr. is over 1010 dilute the urine with an equal amount of water and allow for this by multiplying by two when making the calculation. Albumin may be roughly estimated by boiling the urine in a test-tube and allowing the albumin to settle. The amount of albumin may then be read off as a fraction of the length of tube occupied by the urine; for example, the albumin may be one-third or one-sixth or one-tenth.

IX. If we are dealing with a urine containing a deposit of urates we may apply heat, before filtering; fill a test-tube two-thirds full and heat the upper part of the urine. The turbidity, if due to urates, quickly disappears at the spot where the urine is heated, and this we may contrast with the turbid urine below. If we are dealing with a urine which contains albumin and is turbid from the presence of urates, as we apply heat the urine clears but on continuing to heat the urine again becomes cloudy, due to the precipitation of albumin. Thus in one test-tube we may have from above downwards (1) a cloud of albumin, (2) a portion of clear urine where heat has dissolved the urates, and (3) turbid urine which has not been heated and in which therefore the urates are precipitated.

(*To be concluded.*)

### Hints on How to Start a District Nursing Association.

(*Continued from Page 9.*)

### Subscribers' Tickets.

When there is a fixed charge made for the poor as 2s. and upwards per annum, it is a good plan to provide each subscriber with a member's ticket, which can be had from the secretary, treasurer, or collectors.

This ticket or card should be produced on application for the nurse's services, and the nurse should show the cards to the secretary each week along with her books.

................. NURSING ASSOCIATION.

Member's Ticket.

*Name* ..................... *Address*.....................

Subscription.

| | |
|---|---|
| January ............ | July.................. |
| April ............... | October ........... |

### Rules for the Association.

The rules may be as follows :—

1. The Association shall be called "The ........... District Nursing Association," and shall consist of all subscribers of ......... and upwards, and of the clergy and medical men of the district.

2. The general committee shall consist of subscribers of 10s. and upwards.

3. The executive committee shall consist of a president, treasurer, and secretary, and two or three other members. (Five shall form a quorum.)

4. The Executive Committee shall meet at regular times to discuss business and hear reports.

5. The Secretary shall see the nurse once a week.

6. An Annual Meeting of all subscribers shall be held.

### Rules for the Nurse.

The following rules are suggested :—

1. The nurse shall attend the sick poor and working classes who subscribe 2s. or upwards per annum.

A member's ticket shall be produced on application for her services.

2. The nurse may attend others who are unable to afford a private nurse and who are willing to give a donation in addition to their annual subscription.

Such nursing must be carried out on strictly district nursing lines and must not interfere with the nurse's other work, the poor having the first claim.

3. The nurse shall work under the direction of the medical men and of the executive committee.

4. She shall keep the books provided by the Association and shall report to the secretary once a week.

5. She shall not attend infectious cases (unless special arrangements are made for her other work).

6. She shall not be on duty longer than eight hours if possible, shall attend urgent cases only on Sundays, and shall not sit up at night except in urgent cases.

7. She shall be responsible for all that concerns the patient and the patient's bed, and shall see that the room is kept clean and ventilated.

8. She shall not interfere with the religious opinions of her patients.

9. She shall not accept gratuities.

10. She shall be responsible for all appliances lent out.

11. She shall not be expected as a rule to perform the last offices for the dead, but may do so sometimes for her own patients if her other work permits and she is not a midwife, and if the death occurs in the day time.

These rules work very well in many places, but of course every association must make its own rules to suit local requirements. It is a mistake to make too many; rather have a few well-defined rules to begin with, and, if needed, others can be added later on.

There should be a written agreement with regard to the nurse's salary, the amount of holiday given her in the year, and the length of notice for leaving to be given on either side. In districts where midwifery is undertaken special regulations will have to be made.

### Midwifery and Maternity Nursing.

The nurse should only be allowed to undertake midwifery and maternity nursing if the medical men are in favour of her doing so.

If a nurse be allowed to act as a midwife it should only be among poor people, for if some limitation is not made her services are sure to be unduly called upon; and if she attends people who are quite able to afford a doctor, she may get into trouble with the medical men.

Where the nurse is not allowed to act as a midwife, she may be allowed to attend with the doctor or nurse the case afterwards.

For this branch of work a nurse must have special training, and should hold the London Obstetrical Society's certificate, or such certificate as may be approved by the Midwives Act.

The rules may be as follows:—

1. The nurse will only attend as a midwife among the poor classes.

2. Two months' notice must be given.

3. A charge of not less than 5s. shall be made when no doctor attends.

4. If the nurse attends a case with a doctor, or has to call in a doctor, or nurses a case under a doctor, half the midwifery fee will be charged.

5. The nurse shall attend to mother and child for 14 days.

Having appointed a committee and drawn up some rules, the next step will be to find a nurse. The Association may have to advertise for one, but if the Association be affiliated to a county nursing association, the county superintendent will attend to that.

When seeking for a district nurse every care should be taken to obtain one who is really suited for the work.

She should not only be well trained, but should possess a great deal of tact and common sense, which is almost indispensable in a district nurse. References and testimonials must of course be obtained, and as a rule those from matrons and superintendents are of more value than those of doctors.

### Training.

No matter how small the district and the population, it is best to have a nurse who has had hospital training, and training also in district nursing; and if midwifery is required, special training for that is essential. Ideal training would be:—General hospital, not less than two years; training at a district nursing home, six months; midwifery and monthly nursing, three to six months and L.O.S. Many nurses certainly do very well who have had less training than this; training is not everything, and individual character has a good deal to do with success, while previous work and experience must also be taken as a guide. Special training in district nursing is decidedly a great advantage, for it is utterly different from hospital work, where a nurse has everything needful at hand. A nurse who has had two years in a hospital and six months' training in district work is more likely to be suitable for a post as district nurse than a nurse who has been several years in a hospital and then plunges into district work without any previous experience. For information on district nursing consult "District Nursing," by Miss Amy Hughes, Burdett Series, price 1s., to be obtained from the Scientific Press, Southampton Street, Strand, W.C.

### Village Nurses.

If it is absolutely impossible to raise enough money to keep a thoroughly-trained nurse, a village nurse may be obtained, but this should only be resorted to when the village is really too small and poor to afford a highly-trained nurse. It should be borne in mind that it is in these very villages, often remote from medical help, it is even more necessary that a nurse should be in every way competent. However, it is impossible for some places to raise sufficient money for a fully-qualified nurse and so the village nurse supplies a want, for it is certainly much better to have her than no nurse at all. The village nurse is not a hospital trained nurse, but one who, as a rule, has had six, nine, or twelve months' training in nursing the poor in their own homes. Three months of the time have to be devoted to midwifery, and the L.O.S. examination has to be passed, or an examination approved by the Midwives Act. This training is excellent as far as it goes, but it is very short—no woman can be turned into a "trained nurse" in a few months. In fact, that term can only be truthfully applied to a nurse who has gone through a good hospital course.

*To be continued.*

### An Impression from the Land of the Shamrock.

BY A BRITISH NURSE.

Some of your readers may be interested in a short account of a delightful nursing home in which a friend of mine not long ago had the good fortune to be a patient. In these days one hears such widespread discontent as to the management of many homes, the food, the nursing, and the excessive charges, that I think a pleasant contrast may be afforded by the following brief attempt to describe a very agreeable experience.

First, I should explain in view of the opinion that nurses make the worst and most critical patients, that as both my friend and myself have been trained nurses, with varied experience, our opinion has the merit of being given with knowledge.

The nursing home which has hitherto existed in my dreams only, took actual shape in a stately street in "dear dirty Dublin;" a house not originally intended for patients but which happened to be admirably fitted for their needs. Its wide staircases and shallow stairs made movement and carrying easy, whilst its lofty rooms, wide halls and lobbies seemed full of air and sunshine pouring in through lofty windows. Many of these stately old houses were built in the days when Dublin

AN IMPRESSION FROM THE LAND OF THE SHAMROCK—*Continued.*

was the real capital of Ireland in every sense of the word, built for gentlefolk of means whose axiom was that money was made to be spent, and to whom entertainment and hospitality formed a chief delight. Now adapted to other uses these homes retain their old-fashioned dignity. Here are no mean lines for the tired eye to fret over; in place thereof are exquisitely decorated mantelpieces and ceilings, high and handsome doors with deep mouldings and panellings, and, greatest boon of all, thick walls and floorings which fulfil their primary duty and exclude sound. At the rear of the house a charming flower garden made a bright bit of colour with its tiny greenhouse and well-kept turf. Here, when off duty, the nurses were often at work, weeding, rolling, digging, and doing some really hard work with evident pleasure; they had made out of a waste place a pretty garden, and it struck me that Irish nurses must possess more abounding vitality than London workers. But generalisations are proverbially dangerous, and I recall the fact that the rush of life is not so great on the other side of the Channel. As I observed, however, the garden was a great delight to nurses and patients, and was very helpful in convalescence; moreover, Irishwomen seem to have a specially keen "love of the land" and they certainly have an unusual power of "making things grow."

I could not help being struck by the nurses' wonderful look of health and good spirits, their exquisite complexions, the soft low voices, which were all factors in "making for" convalescence. As Mr. Austin Dobson has said "the Irish may be sad but they are seldom serious," and this I conclude is the cause for the bubbling merriment which comes so near the surface, for the amusing side of life is always very close; there are certainly worse mottoes in a "worriting" age than "Be aisy; and if you can't be aisy, be as aisy as you can."

Amongst the other and more material factors at work in the home were the delicious food, the well served meals, the little trays covered with finest Irish linen, ornamented with delicate drawn thread work, all of spotless purity, and the fine china. Of the quality of the food and the cooking I cannot say more than that they were perfect; milk, butter, and eggs were brought from a larger institution and were most excellent, with true country freshness.

I visited the home constantly, giving a great deal of trouble as is the nature of "patient's friends" all the world over, but never was I allowed to feel anything but a welcome guest, from the moment when the kind and brilliant surgeon, then a stranger, said to me just before my friend's operation, "I want you to feel in this room just as in your own hospital," till the last good-bye was said and the last kind act of care accomplished for the patient in whom I was so much interested. To sum up the essential advantages which struck me in this home above others I have known, both as nurse and patient, were:—

1. The complete absence of red-tape, a most soothing omission to anxious friends and relations; the acknowledged tone that this was a real home, *i e.* where the patient would naturally want to see her friends, and where all intercourse should be made as easy as possible. The spirit was in fact most human and the effort seemed always to be "how pleasant can we make this unpleasant experience?" for such any sojourn in a nursing home must ever be to some patients.

2. No fancy of the patient was discouraged unless really harmful, and any change in the arrangement of furniture, etc., was made with a pleasant grace, the patients' desires were in truth of first importance. This is as it should be, where other patients are not present to be made jealous by "fadding," as is the case in a large ward, but private nurses in homes do not always, I think, take this into consideration sufficiently.

3. Not one word regarding other patients reached the various cases. Instant dismissal followed any breach of the strict rule of "No gossip allowed;" a wise and good rule both for nurses and patients, and carried out in the spirit and letter as I can testify, although from outside sources I happened to know, at the time, that the home had many anxious cases in it.

4. The standard of outdoor conduct, behaviour, and neatness was kept up by the fact that the name of the home was embroidered on the nurses' uniform and cloaks in a small but distinct circle; further that the uniform was registered, and the nurses were instructed to inform the heads of the home if they should happen to see anyone wearing the uniform or a copy of the same. I should add that the nurses at intervals worked in the hospital attached or in private nursing, but it was considered a privilege to return to the home, so excellently was it managed. The fees were most moderate and inclusive; only good management could have made this possible, as Dublin rents are by no means low.

And now having summed up my impressions, I should like to explain under what body this most admirable home is organised. It is that of a trained and devoted order of nuns, having under them trained lay nurses of their own faith. They have a hospital close to the home in which there is a trained lay matron, and in which probationers are trained, but the latter are not allowed even to visit the nursing home, which has a covered way in connection with the hospital.

The nun who was in charge of the nursing home had indeed responsible duties, and led a life which to the outsider seemed difficult and exhausting. Up at five every morning, seldom in bed before eleven, always on her feet, and constantly mounting the long flights of stairs, her presence and personality seemed to me the motive power on which the beautiful and harmonious spirit of the home depended. Never once did I see her without a bright and friendly smile; her face and picturesquely (though most practically) garbed little figure seemed to radiate peace and health. How tired she sometimes looked all the same! Yet in spite of constant supervision and tireless energy, she still had ever ready an amusing story, a hearty laugh, a kind thought, or sometimes silent sympathy for each individual patient, as their needs varied. I learnt something of the secret of success in that home when I found that not one of the many little trays reached a patient's room without the Sister's observant eye "passing it in review;" her personal knowledge of the different cases, their likes and dislikes, were all noted "for remembrance." Sister always knew exactly how much a patient had eaten or left. The actual cooking was in the hands of a lay sister, and was excellent.

I could not but admire Sister's beautiful countenance, with its unmistakable revelation of the fine and noble spirit that lay behind it, but I admired yet more her character, as I saw the love and willing service she inspired, her marvellous patience with forgetfulness (for I admit that Irish "colleens" have often short memories), and her daily life of finely inspired and thorough work for which years of training in every detail of nursing and household management have so well prepared her. Finally, I summed up my impressions in the words of Dr. Weir Mitchell, in his recent lecture on the education of nurses. He says:—"I have often wanted to unite the entire self-devotion of the sisterhoods with the perfect training of the secular nurse. I have seen and admired the union of perfect training and high sense of religious duty combined in the lay nurse, but it is rare, very rare." Yet I found it here.

### The Victorian Trained Nurses' Association.

BY AN AUSTRALIAN MATRON.

That the establishment of the above association in the Colony of Victoria is a step in the right direction is a fact that must be conceded by all. Training schools at the larger metropolitan and provincial hospitals have now been established quite long enough to ensure such a steady supply of intelligent, well-trained and up-to-date nurses as to make it no longer a necessity for doctors and families suddenly afflicted with illness in the house to have to fall back upon nurses who have had no hospital training whatever, nor even hospital experience. That the best efforts of medical skill may be frustrated by an incompetent nurse is a truism, but when fees are good—as they are in this country—it is unfair to those who have to pay them that the nurse employed should not be in all respects thoroughly competent.

### Needful Concessions to Uncertificated Nurses.

The initiation of the movement was necessarily surrounded by many difficulties, and the organisers, in order to ensure success, found themselves obliged to make concessions which at a later stage of the association's existence might not have been considered advisable. Rule 21 reads as follows:—

21. Any nurse also, who can show to the satisfaction of the Council that she has been engaged for not less than three years in the *bonâ fide* work of medical and surgical or special nursing, either in hospitals or in private nursing, and can produce certificates of competency and good conduct satisfactory to the Council, from at least three reputable medical practitioners as to her attainments and repute, shall be eligible for registration up to June 30th, 1902, subject to the approval of the Council. Provided always that the Council shall have power in any case to direct that any candidate under this rule may be examined in practical nursing by three examiners appointed by the Council, and provided further, that in the case of nurses registered under this rule the qualification in the register shall stand as "admitted by the Council under the provisions of rule 21."

Many nurses registered under this rule were nurses of very good practical hospital experience, but in their time, at the principal hospitals here training schools were not then in existence, and of course no certificates were issued, and it would be depriving these nurses of their means of living after years of thoroughly good work to exclude them.

### Multiplicity of Recognised Training Schools.

It will be seen by the register that nearly all the hospitals in Victoria are in future to be recognised as training schools, but in a colony with a population of less than a million and a quarter like Victoria, I do not think this quite necessary and there appears to be a danger of the colony having too many nurses ere long. This may, and probably will, have the effect of bringing down fees, as the annual output will be considerably increased. Moreover, many of these hospitals on the list are very small—one has as few as nine beds and others have under twenty. Surely the educational advantages of such hospitals cannot be very great, and possibly at no very distant date the Association may find it advisable to amend its rules, so that only general hospitals with not less than 50 occupied beds as a yearly average of "hospital patients" shall be recognised as training schools. I use the words "hospital patients" advisedly, because some of the institutions registered as training schools are only partly hospitals and partly benevolent asylums, and there are instances in which the yearly average number of beds occupied as hospital beds amounts to only about one-half of the total number, with which the institutions are credited in the list of training schools. In one case it does not amount to even one-fourth.

### Certificates.

With regard to the certificates issued by the hospitals themselves, I think that there ought to be some sort of classification of the nurses, according to ability, and that the certificate should show the standing of the holder, otherwise all nurses stand on the same level and the least competent receive the same as the most competent. This system would be of valuable assistance to the holders in obtaining hospital appointments.

### The International Hospital in Venice.

BY A CORRESPONDENT.

How many of those who lounge in a gondola in the moonlight of a Venetian evening watching the red gold of the gas lamps reflected on the silvery surface of the lacoons and canals, pause to think of the wants of the numerous sailors on English vessels which, amongst other commodities bring, from British shores, the coal to make the gas for the "Queen of the Adriatic." Yet these men are exposed to many chances of ill-health and accident, and hitherto when overtaken by illness they, as well as all other foreigners, have had to endure the isolation and strangeness of a foreign hospital. The authorities of the Ospedale Civile, Venice, have been amongst the first to realise the good effect produced upon patients by suitable and congenial surroundings, and the large wards of a foreign hospital are worse than a desert to the invalid who is cut off by ignorance of the language from any communication with doctors, attendants, or his fellow patients. Now this state of things need no longer exist, for an unpretentious but thoroughly practical "International Hospital" has been opened in Venice. The ceremony was performed last week by her Royal Highness Princess Christian, accompanied by Princess Victoria. They were received by Lady Layard and the other members of the Anglo-Saxon community in Venice, through whose exertions the little house and garden in the Fondamento Rioddla Croce have been secured for the use of the institution during a period of five years, with the option of purchase on very advantageous terms ere the lease expires.

At present the accommodation is limited to one ward of five beds, a small operating-room, and the necessary sitting-rooms, kitchen, and offices. The whole is in charge of Sister Clare, formerly of King's College Hospital, London. After the opening ceremony those invited to the gathering were free to wander over the premises up to the second floor where the sisters' rooms are situated, and into the little garden, where refreshments were served in the former plant shelter, the rafters being festooned with flags of all nations, lent by the skippers of one or two colliers discharging at the Punto Franco. The Prefect of Venice, with representatives of the governing body of the Ospedale Civile and the consuls of most of the foreign nations trading on the Adriatic were present, as well as doctors and surgeons of various nationalities, all meeting together on the common ground of charity. In this way they followed the example set by our own Royal Family and the Queen-Mother Margherita of Italy, who has already graciously contributed to the fund for meeting the initial expenses of the undertaking. The year's outgoings at present are estimated at £250. To meet this outlay there are interest on funded moneys, £30; annual subscriptions promised, £65—total, £95, leaving the balance of £155 to be met by the donations of friends and the co-operation of the Boards of Trade of each nation whose vessels call at this port. In time other rooms of the house will be arranged for the use of patients, who are expected to average about fifty per annum of British tars alone! But until the financial outlook is more certain the committee have wisely determined to proceed cautiously.

### Bulbs for Hospital Decoration.

BY AN IRISH CORRESPONDENT.

One of the greatest helps to an invalid towards getting back health and strength is to learn to look on the bright side of things. What could be better for this purpose than flowers with their charming tints and fragrant smell, giving the patients both amusement and pleasure in watching them growing in form and beauty every day? But in order to have flowers in the spring now is the time to begin. For the spring decoration of hospitals bulbs are the most suitable, as their smell is not too oppressive nor their colours too vivid. Some, however, may think that they have neither time nor the right soil or place to grow them in. This is a mistake. Bulbs as a general rule do not require any particular soil, for they hold within themselves enough food material to last while they are in bloom. The bulb really is the storehouse or larder from which the flowering spike and leaves take their nourishment as they require it. As regards the proper place to cultivate them in they will grow in any place indoors but do best near the light, after they have been started in the dark or under ashes. When once planted nothing has to be done to the bulbs except watering when needful, and in this respect they are a contrast to delicate ferns and palms which need daily attention and care as well as a thoughtful eye upon them. However the best results will be obtained by those who place their bulbs, when planted, under ashes or paper in a cool place, keeping them dark until the pots or boxes are well filled with roots when they should be exposed to the full sunlight.

### The Question of Planting.

For this sort of decorative work they should be planted in boxes, bowls, saucers, jars and ornamental pots in the best soil obtainable, placing a little silver sand at the base of each bulb, making them firm but not covering up quite all the top of the bulb. Of course cocoanut fibre can be used but it is the most expensive material, and money to most nurses is more or less of a difficulty. Nurses and sisters have to supply many things besides flowers, therefore I should like to add that the generous public could not do a greater act of kindness than to send bulbs, even if they are their old ones from last year, to the hospitals to be grown. But if the nurses have to buy, and to buy economically, this can hardly be done by taking up one of the very elaborate catalogues sent out by different firms, which are all very well for the professional gardener, but to the amateur a maze of names meaning very little. I give a list of a few good varieties in the hope that they may be useful.

### Hyacinths.

Of hyacinths there are two kinds:—(1) Roman hyacinths, which are the first of all bulbous plants to flower, and which do best in pots, jars or boxes, but are not suitable for window-boxes. (2) Ordinary hyacinths, either for boxes or jars, which are specially made for them, and are especially useful for window-boxes, remembering to choose, if possible, good varieties.

### Narcissi (Daffodils).

(1) The first of this section are the polyanthus narcissii, which are suitable for pots, boxes and bowls; (2) Princeps maximus; and (3) Cynosure; both being single-trumpet daffodils, come in well for pots, boxes, bowls, saucers, etc., whereas the double-trumpet daffodils, as a rule, are too heavy for bowls and saucers. (4) Poeticus Ornatus (Pheasant Eye)—This is one of the most charming of all daffodils, which has many advantages, being inexpensive, slightly fragrant, pretty in colour, and suitable for all purposes.

### Tulips.

These bulbous plants are also very useful for decorative purposes, especially nowadays when there are so many different shades of colour, differences of shape, and inexpensive varieties. But here I will only mention a few varieties to go upon, which are specially good, namely—Scarlet Duc, La Reine, La Candeur, Yellow Prince. In addition to these, there are the ever-charming little snowdrop, the first sign of spring, with its companion, the crocus, of many shades of colour. The snowdrop and the crocus will be found to be especially suitable for acting as a groundwork for the others. Nothing looks better than a bowl with a groundwork of snowdrops from which stands the stately pheasant eye narcissii, one acting as a foil for the other, or again the crocus with cynosure as its leading flower. But all details I will leave to the grower, allowing her full scope for her imagination and individual taste, which will be well rewarded if the few directions are as far as possible carried out. Then when the plants break forth into blossom and fill the wards with their glorious tints and smells, the nurse will be able to point out to her patients how Nature furthers her ends, not stopping until the goal is reached, no matter what difficulties arise in the way, showing them how much the more should they strive to regain the health and strength which was originally given them to enable them to carry out their allotted work in life.

### For Elderly Nurses.

A nurse who has turned forty and has not obtained a post as matron occasionally feels herself a veteran who lags superfluous on the stage. If she is still working in a hospital she may find the long days in the wards too hard for her. If she is doing private work she knows that in a few years her cases will begin to get scarce. Young nurses are in fashion. They are supposed to be stronger, more adaptable, more cheerful. This is not always so, but such is the popular notion. Patients and their friends feel that they must "consider" the elderly nurse more than a younger woman, and—not from any unkindness, but simply from the fact of illness being in the house—they don't want to have to show special consideration. Even maternity work, which used to be the preserve of the middle-aged woman, is now being invaded by younger rivals, and young mothers find the young nurse more companionable during their convalescence than an older one. For many reasons there comes a time when a nurse, still able to do good and useful work, finds herself "laid on the shelf." For such women an opening may be found in acting as foster-mother to children who are under the care of Poor Law Guardians. More and more parishes are seeing the wisdom of bringing up the children under their care in small families. Sometimes they board them out in artisan households, but the number of households which fulfil the requisite conditions is limited. The guardians must see that they do not send their wards to a place where they will be made drudges to the children of the family. Thus some unions vary the boarding-out system by one of scattered homes. These homes are simply houses in the artisan quarters of a town or village, hired for the most part, though sometimes the union has to build suitable ones, and occupied by six or seven children of different ages—so as to simulate as nearly as may be the ordinary conditions of family life—who are put under the charge of a foster-mother. The foster-mother receives a salary of from £20 to £25 per annum, in addition to board and lodging. The elder girls of the family, and the boys also, give some assistance in the household work, but no hired help is given. The position is a humble one, but it is one of great importance, as on the way these children are brought up it depends, in all human probability, whether or not they become paupers, thriftless and useless, if not absolutely wicked, as in most cases their parents have been, or useful and capable citizens. The training in method, in absolute cleanliness, and in noting the signs of disease which nurses receive should make them fit to fill such a post ideally. Of course, they would need to be kindly women, patient with children who often have inherited weaknesses (moral and physical), yet firm as well as kind. But here, too, long experience of invalids and their ways should form a good training. A woman who would despise her nurselings would certainly be useless, but one who could pity and grow to love them would find the work interesting and ennobling as well as useful. And assuredly such a post is better than growing old with the feeling that one's power of working and chances of work are slipping away from one.

### Wants and Workers.

Will anyone kindly send old white rags for the use of a very bad cancer patient to Mrs. Foley, care of Mrs. Cooper, 7 Cheetham Place, Cheetham Hill, Manchester.

### TRAVEL NOTES AND QUERIES.

By our Travel Correspondent.

Passage to Durban (T. L. S.)—We do not reply by post unless 2s. 6d. is sent for our Convalescent Fund, but I hope this will catch your eye. Write to Messrs. Cook and Sons, Ludgate Circus, E.C., and ask them for full particulars of the sailings to Durban from London, mentioning that you want the cheapest line, and also state at what time of the year you want to go. They will send you special papers with everything you want to know.

Visit to the Holy Land (Matron).—It was not in our paper that you saw the notice you speak of, but I can tell you of a tour to the Holy Land starting about that time. Write to Dr. Lunn, 5 Endsleigh Gardens, W.C., and ask for particulars. I am not sure at what date he starts, but I fancy in January. We do not answer by letter unless 2s. 6d. is sent for our Convalescent Fund, but if you write me again I can answer the following week in this column.

Naples for a Long Stay (Kathleen).—I am so glad the trip proved a success, and I have no doubt you feel the pleasure was worth all the extra money. It is one that remains. Now as to Naples: (1) cheapest route Newhaven and Dieppe, Paris, Genoa, and Rome, second class £7 0s. 7d. (2) There are very considerable changes in the temperature in the course of 12 hours very often, but that, for a healthy person who exercises ordinary care, is not dangerous. The winter is on the whole mild, but extreme cold is experienced at night in January. April and May are delicious months in Naples. The summer is hot, but the proximity of the sea prevents the extreme sultriness often felt in Southern Italy. It is much better in its sanitary arrangements than it was a few years since, and I think there is nothing to fear on the score of healthiness. (3) You would want much the same outfit as would be needed for the same period in England, supposing you were unable to renew garments. Woollen underclothes are absolutely essential at all seasons, but you can have them of varying thicknesses. Remember that the variable temperature necessitates somewhat warm clothes. (4) The shops are very good, but not cheap, and I should certainly not recommend you to buy blouses there, you would pay nearly double the price of the same goods in England. Take what you want with you, but do not overdo it, one usually takes too much. (5) All luggage on Italian rails has to be paid for except what you carry in your hand. The money is very simple, it is usually paper in 1 or 5 or 10 lire notes up to any amount. A lira is the same as a franc, but the rate of exchange is favourable to us, for you get usually 27 lire for a sovereign.

### Everybody's Opinion.

### CHANGES AT KING'S LYNN HOSPITAL.

"Veritas" writes: Concerning your note on King's Lynn Hospital, I am one of the numerous nurses who left that institution recently. I can say from my own personal experience that what you state is true, and also that many nurses would be glad to hear that the board of the King's Lynn Hospital have investigated into the cause of the constant changes in the nursing staff.

### THE NORTH-WESTERN POOR-LAW CONFERENCE AND NURSING.

"Mr. H. Meadows" writes from Altrincham: There seems to be some little misconception about my paper on Workhouse Management, at the North-Western Conference. I did not for a moment intend to minimise the importance of training and certificates for nurses, but as the cases they have chiefly to deal with require kindness and care rather than great skill, I did lay particular stress on character. As regards the superintendent nurse, I am also misunderstood to some extent. I distinctly stated that the nurse should have sole control of the nursing and also of her assistants, and that she should not be interfered with by the matron; but as regards the latter, I urged that it was derogatory to the dignity of a worthy matron to have her liberty so curtailed that she was not at liberty to visit any part of the establishment whenever she desired.

### ANSWERING ADVERTISEMENTS.

"A Matron" writes: I think that my experience was in a measure even worse than that of your correspondent "J. B." Three years ago, when a sister in a large general hospital, I applied for a post as matron of a small special hospital near Liverpool. Complying with conditions, I forwarded photograph and copies of testimonials, enclosing a stamped addressed envelope for their return. In answer to a letter of inquiry (*re* my people) I forwarded an original letter from the family physician, who had known me all my life. This was evidently satisfactory. In the course of a few days I received a notification that I was a selected candidate, and the date of the interview would be forwarded later. I waited patiently, and in the interval was offered the matronship of a cottage hospital, which I kept in abeyance, holding myself in readiness for a summons from Liverpool. Judge of my surprise when, two weeks later, I saw in The Hospital that a matron had been appointed. I never had any explanation, nor were my photograph and testimonials returned, in spite of my writing for them and enclosing another stamped addressed envelope. The original letter was also kept, and, by my delay, I missed the cottage hospital appointment also. The moral is, obviously, never to send original testimonials unless absolutely obliged, and be prepared to sacrifice any copies that may be forwarded.

### NUNS AS NURSES.

"A Trained Nurse" writes: "Cecilia" misses the point of Mr. Wyndham's statement. It was that as nuns the sisters at the Granard Workhouse "did not care to perform, and ought not to be asked to perform, some parts of hospital work." He did not, as a layman, attempt to define the limits of a nurse's duties. I agree with "Cecilia" that in many hospitals "there is a great diversity of opinion as to how far a nurse's duty extends," but this is in regard to her services to male patients, and therefore has no bearing on the present case in which, if I am right, the patients were women, and the cases the nuns refused to nurse were gynæcological or midwifery ones. I repeat that no hospital nor workhouse would retain the services of a nurse who declined to perform this part of her nursing duties, and therefore, in judging the nuns from a professional standpoint, I should say they had failed in their duty as nurses if they did so refuse. And if either nurses or nuns put their own likes and dislikes before the needs of their patients, and limited their services to them to what could be legally exacted under their agreements, they would fail in their duty as religious women, which duty seems to me to lie in trying unostentatiously "to do the will of the Father which is in heaven." "Cecilia's" contention, that "everyone is not bound to do whatever anyone else can be made to do," is beside the mark, there being no compulsion in the case, trained nurses, who were perfectly free agents, being engaged to undertake the nursing duties the nuns would not perform. I have never during ten years' nursing experience in civil and military hospitals seen a nurse other than gentle and reverent in the presence of death or of an unconscious patient. As I have never heard of a nurse's presence being required at a post-mortem examination I cannot say how her attitude in such a case might compare with that of a nun, and I should think any comparison as to the nurses' and nuns' knowledge in the discussion of their cases would probably be considerably to the advantage of the nurse. I would like to remind "Cecilia" that a sanctimonious pose is a matter quite apart from religion, and a high moral tone is not by any means the rarity she imagines it to be in large hospitals.

### Novelties for Nurses.

By Our Shopping Correspondent.

### A NEW ANKLE-SUPPORT.

The American Shoe Company, Regent Street, are selling a new ankle-support, which deserves a trial, in place of the simple method of bandaging. The ankle-support is made of black sateen or coutil lined with white, and passing under the instep it is laced at the side, thus remaining firmly fixed in position. The sides are stiffened so as to secure effective suppport. The supports are neatly and durably made.

### Appointments.

Axbridge New Infirmary.—Miss Charlotte Tuck has been appointed superintendent nurse. She was trained at Stapleton Infirmary, Bristol—where she has since been staff nurse—and Bristol Royal Infirmary. She holds the L.O.S. certificate.

Camberwell Infirmary.—Miss Lucy Creemer has been appointed superintendent night nurse and Miss Florence Boyce, M. B., sister. Miss Creemer was trained at Camberwell Infirmary and Miss Boyce at Mile End Infirmary.

Great Yarmouth Workhouse Infirmary. — Miss Dorothy C. Howlett has been appointed superintendent nurse, and Miss Caroline Berridge charge nurse. Miss Howlett was trained at Aston Infirmary, where she was subsequently charge nurse. She has since been sister at Bethnal Green Infirmary, night superintendent at Fulham Infirmary, and superintendent nurse at Sudbury Union Infirmary. Miss Berridge was trained at Holborn Union Infirmary, where she was afterwards staff nurse. She has since been staff nurse at Wandsworth and Clapham Union Infirmary, and at Berkhamstead Union Infirmary. She has also done private nursing.

Isolation Hospital, Carlton, near Worksop.—Miss Blanche Drake has been appointed nurse-matron. She was trained at Essex and Colchester Hospital, and has since been on the private nursing staff of the York Home for Nurses, nurse at the Sanatorium, Scarborough, and nurse-matron at the Isolation Hospital, Sicklinghall, Withorley.

Thos. Knight Memorial Hospital, Blyth, Northumberland.—Miss Mary E. G. Brough has been appointed matron. She was trained at Hartlepools Hospital, where she has since been charge nurse.

### The Nurses' Bookshelf.

Materia Medica for Nurses. By John E. Groff, Ph.G., Apothecary in the Rhode Island Hospital. Second edition. Revised and re-written. (Philadelphia: P. Blakiston, Son and Company. Pp. 169. 1903. Price $1.25 net.)

Although this little work has apparently met with approval in the land of its birth, it is hardly likely to prove popular either with doctors or nurses in this country. In our opinion it is not only an undesirable, but even an actually dangerous book to place in the hands of the average woman who takes up nursing as a profession. It contains far too much to be useful and not enough to secure safety. Even from the outset we are opposed to the aim of the author, for in the second sentence in this guide to "substances and means used in curing disease" he says: "The nurse must, in order to afford the intelligent aid to the physician which is expected of her, make herself familiar with the names and nature of the principal drugs and chemicals in use; the medicinal properties, physiological action, uses and doses," and he goes on to add, "she should also know the names and uses of the various preparations made from them." We no more approve the author's contention than we admire the form of grammatical construction in which he has expressed it. The work contains much concerning weights and measures, the metric system, dosage, definitions of pharmaceutical and chemical terms, and a classification and description of drugs. There is also a chapter on "new remedies" into which various proprietary preparations have been admitted. A short section on toxicology does not seem out of place, although it is likely to prove practically useless in counteracting the evils likely to be wrought by any nurse who attempts to follow for herself the directions given in these pages.

The Mystery of Being: the Story of Life for the Use of Mothers of Boys. By Ellice Hopkins. (London: The Walter Scott Publishing Company, Limited. Price 6d.)

Professor Sully in his fascinating book on "Children's Ways" shows how the growth of things affords one of the most stimulating of childish puzzles: "the origin of babies and young animals furnishes the small brain with much food for speculation." Unfortunately only too frequently the mystery of being is unmasked by rude and ignorant hands, and Nature's methods allowed to furnish material for the lewd and lawless. But to present the story of life to boys and girls in purity and simplicity and yet with scientific truthfulness is a task of immense difficulty. Ellice Hopkins seeks in this little work to impart pure knowledge on the subject of life and birth to the young. We quite agree with the method adopted, and strongly believe that it is better to teach a boy these matters by reference to what we understand by "natural history" rather than "human physiology." It is not intended that this brochure should be put off-hand into a boy's hands, but rather form a basis from which wise parents while reading it to their children, may impart true instruction. But we think a parent sufficiently wise to expound the truth as is here so beautifully shrouded could well dispense with such a work altogether. There is much in the book that is excellent and to some few wise mothers it is likely to prove of much service. Teachers may well learn something from its manner and methods and wise suggestions. But surely scant wisdom and great inaccuracy is manifest in speaking of the mother's body as "rent and torn to give entrance into life." The author has attempted a peculiarly difficult task, and has succeeded in obtaining no small measure of success. We have no hesitation in commending the consideration of this booklet to all thoughtful parents and conscientious teachers.

### Echoes from the Outside World.

### Movements of Royalty.

The King returns to London this week, also the Prince and Princess of Wales. The Queen and Princess Victoria arrived at Darmstadt on Tuesday in order to be present at the marriage of Princess Alice of Battenberg and Prince Andrew of Greece. They met with a very cordial reception from the people whilst driving through the streets. The civil marriage took place on Tuesday, and the religious ceremonies on Wednesday, in the presence of many other illustrious personages including the Czar of Russia.

### The Political Crisis.

There was a great gathering of Unionists at Sheffield last week under the auspices of the National Union of Conservative and Constitutional Associations, the interest of the proceedings centreing in the speech delivered by the Prime Minister at the Artillery Drill Hall on Thursday night. Mr. Balfour dealt exclusively with the tariff question, and invited the people of this country to give the Government, from whatever party it might be drawn, the freedom of negotiation of which they had been deprived. He thought that public opinion with regard to closer fiscal union with the colonies was wrong. Hostile tariffs had deviated our industries into channels into which they were never intended to go. Common justice and common fair treatment were all they asked for, and if they did not get them they would take their own measures. The policy which he outlined was approved at the Conference on Friday morning, and the Conservative party have therefore formally pledged themselves to the support of retaliatory duties. At a luncheon to the Conservative agents on Friday Mr. Balfour, after paying an eloquent tribute to Mr. Chamberlain as "the greatest colonial minister whom this country had ever seen," announced, with much regret, that Lord Milner had not found it possible to accept the office because the High Commissioner held that there were problems of great difficulty and delicacy to be dealt with in South Africa with which he might be able to deal more effectively on the spot than he could from Downing Street.

### New Ministers.

On Tuesday it was officially intimated that the Duke of Devonshire had resigned his position as Lord President of the Council. On the same day it was also announced that Mr. Austen Chamberlain succeeds Mr. Ritchie as Chancellor of the Exchequer; that the Hon. Alfred Lyttelton becomes Colonial Secretary in the place of Mr. Chamberlain; that Mr. Brodrick replaces Lord George Hamilton as Secretary of State for India; that Mr. Arnold-Forster, Secretary to the Admiralty, succeeds Mr. Brodrick as Secretary of State for War; that Lord Stanley, Financial Secretary to the War Office, enters the Cabinet as Postmaster-General instead of Mr. Austen Chamberlain; and that Mr. A. Graham Murray, Lord Advocate of Scotland, succeeds Lord Balfour of Burleigh as Secretary for Scotland. Mr. Lyttelton has never held office before, but he has been actively engaged in affairs connected with the Colonies, and was chairman of the Commission which visited South Africa to investigate the subject of concessions granted by the Transvaal Government. He is a nephew of the late Mr. Gladstone.

### Mr. Chamberlain's Campaign.

On Tuesday Mr. Chamberlain opened at Glasgow his campaign in exposition of his fiscal purposes, obtaining an enthusiastic reception from a vast audience in St. Andrew's Hall. The interest, which was maintained throughout, culminated in the outline he gave of his plans for stimulating the commerce and industry of this country and the Empire. He did not propose any tax on raw material, but a low duty, not exceeding 2s. per quarter, on foreign corn, no tax on maize, a tax on foreign flour, a 5 per cent. duty on foreign meat and dairy produce, with the exception of bacon, a substantial preference to Colonial wine and fruit, and the remission of three-quarters of the duty on tea and half that on sugar, with a corresponding reduction on coffee and cocoa. Upon the assumption that they paid the whole of the new taxes, the agricultural labourer and the artisan would be half a farthing a week better off. But he believed that the new taxes would be paid by the foreigner. Mr. Chamberlain estimated that the loss to the Exchequer under his scheme would be £2,800,000 per annum, but he proposed to make that amount up by a moderate duty on manufactured goods which would yield £9,000,000 a year, the balance of revenue being used for the further reduction of taxes on food, and other taxes which press hardly upon the people.

### The Pope's First Encyclical.

The first Encyclical issued by Pope Pius X. on Saturday evening is a remarkable document. His Holiness states that it was contrary to his own wish that he was elected, knowing himself to be unworthy to succeed Pope Leo XIII., and also because he is dismayed at the present condition of human society. He declares that his sole programme will be the revival of the Christian life, and that he will have no secret tendencies, no secret aims, and no party preoccupation. The Pope invokes the co-operation of the Bishops, deplores the war against God which is everywhere being waged, and as a remedy says that the customs of the Church must be remembered and Christian instruction must be imparted. He approves the formation of Catholic societies which set the example of a Christian life, and he affirms that works of charity must be performed without thought of self or seeking for earthly gain.

### Miss Marie Hall's Recital.

A large and admiring audience assembled at Queen's Hall on Saturday afternoon to hear Miss Marie Hall, and were provided by her with liberal fare. First she dealt with the exceeding difficulties of Paganini's D Major Concerto in the masterly manner with which those who have seen much of this young artist have grown to associate her name, and revealed again her absolute accuracy of intonation and her ease in execution. Her first piece was followed after too short a respite to allow of much rest, by Vieuxtemps well-known Concerto in E Major, and in this Miss Hall showed unmistakable signs of the strain of her previous performance. However, she had quite recovered her usual form by the time she came to the "Introduction and Rondo Capriccioso" of Saint Saëns, and her hearers gave practical demonstration of their appreciation of her talents.

### Madame Duse in London.

Madame Duse has returned to London again for a short spell, and those who have once seen her act will doubtless make a point of visiting the Adelphi Theatre, for she still remains one of the greatest of living actresses. The fact that she and all her company speak Italian is merely a trifling drawback, for in some mysterious manner she is so thoroughly the character she represents that she communicates her feelings and her personality to her hearers. Madame Duse appeared the first evening in *La Gioconda* because the examiner of plays refused to permit her to produce *La Città Morta*. It is a sombre tragedy written especially for the Italian actress. La Gioconda is a beautiful model who lures the sculptor Lucio Settala from his allegiance to his wife Silvia, the rôle undertaken by Madame Duse. In the third act, in a fit of rage, La Gioconda pulls a statue down and it falls on Silvia, crushing her hands so badly that she can never use them again. The sadness of the fourth act under these circumstances can be imagined especially as Silvia does not regain her husband's love.

### For Reading to the Sick.

"THE BOND OF CHARITY."

Our fellow-travellers still
Are gathering on the journey! the bright electric thrill
Of quick instinctive union, more frequent and more sweet,
Shall swiftly pass from heart to heart in true and tender beat.
And closer yet and closer the golden bonds shall be,
Enlinking all who love our Lord in pure sincerity;
And wider yet and wider shall the circling glory glow,
As more and more are taught of God, that mighty love to know.

*F. R. Havergal.*

---

Lovest thou God as thou oughtest, then lovest thou likewise thy brethren:
One is the sun in heaven! and one, only one, is Love also!
Bears not each human figure the god-like stamp on his forehead?
Readest thou not in his face thine origin? Is he not sailing
Lost like thyself on an ocean unknown—and is he not guided
By the same stars that guide thee?

*Longfellow.*

---

Only in looking heavenward, not in looking earthward, does what we can call Union, Mutual Love, Society begin to be possible.—*Carlyle.*

---

The Life of grace and truth; that is, the life of Christ, and therefore the Life of God. The Life of grace—of graciousness, love, pity, generosity, usefulness, self-sacrifice; the Life of truth—of faithfulness, fairness, justice, the desire to impart knowledge, and to guide men into all truth. The Life, in one word, of charity, which is both grace and truth, both love and justice, in one Eternal Essence. That is the life which God lives for ever in heaven. That is the one Eternal Life, which must be also the Life of God. For, as there is but one Eternal, even God, so is there but one Eternal Life, which is the life of God and of His Christ. And the Spirit by which it is inspired into the hearts of men is the Spirit of God, who proceedeth alike from the Father and from the Son.

Have you not seen men and women in whom these words have been literally and palpably fulfilled? Have you not seen those who, though old in years, were so young in heart that they seemed to have drunk of the fountain of perpetual youth—in whom, though the outward body decayed, the soul was renewed day by day; who kept fresh and pure the noblest and holiest instincts of their childhood, and went on adding to the experience, the calm, the charity of age? Persons whose eye was still so bright, whose smile was still so tender, that it seemed that they could never die? and when they died, or seemed to die, you felt that they were not dead, but only their husk and shell; that they themselves, the character which you had loved and reverenced, must endure on, beyond the grave, beyond the worlds, in a literally everlasting life, independent of nature, and of all the changes of the material universe.

*C. Kingsley.*

---

And thus at times, as Christians talk
Of Jesus and His Word,
He joins two friends amid their walk
And makes, unseen, a third.
And oh! how sweet their converse flows,
Their holy theme how clear,
How warm with love each bosom glows
If Jesus be but near!

*Grinfield.*

### Notes and Queries.

**FOR REGULATIONS SEE PAGE 21.**

*Home.*

(17) Would you tell me where a young girl can be received who is dangerously ill of pulmonary tuberculosis? I have applied to the Friedenheim Home, but they are not able to help. Her relatives are working people and have no time to attend to her properly, but would contribute to her maintenance.—*A. E. S.*

Try the Free Home for the Dying, 29 North Side, Clapham Common, S.W.; Nazareth House, Hammersmith; St. Marylebone Home for Incurables, 61 Weymouth Street, Portland Place, W.

*Midwives Act.*

(18) 1. Will you kindly tell me when the Midwives Act comes into force so that unqualified women must cease to work for gain. 2. When a maternity nurse is engaged four months before the case is expected, and when the event occurs a month too soon and she is unable to be present, is she entitled to recover any part of her fee?—*E. M. B.*

1. The Act is already in operation; it comes fully into force in April 1905. 2. It is usual for the nurse to receive half fees. N.B.—When you book a case you should ask for a written agreement to this effect.

*Workhouse Matron.*

(19) How long would it take a domesticated young woman to train as assistant workhouse matron, and how could she obtain the instruction required?—*J. R.*

A year or longer. Advertise for post under matron, stating that the girl is desirous of training.

*Indian Nursing Service.*

(20) I wish to know if it is necessary to be trained in a London hospital for the Indian Nursing Service; and if I should find it difficult to obtain training at Guy's?—*S. E. J.*

No, nurses trained at approved hospitals in the provinces are eligible. The matron of Guy's will give you particulars about the training at that institution.

*Patent.*

(21) Could you advise me how to patent the enclosed pattern of a massage wrapper? I find that it is a thing very much needed.—*Nurse Nellie.*

Apply to the Patent Office, 25 Southampton Buildings, W.C.

*Registration of Trained Nurses.*

(22) Will you very kindly tell me if there is any means of registering trained nurses, and what the qualifications required are?—*L. S. C.*

There is no registration of nurses in England.

*Hospital Training.*

(23) I should be much obliged if you will tell me if 18 is too young to enter a hospital, or infirmary, as a probationer for nurse. —*M. L. H.*

Yes, five years too young.

My age is 42, am I too old to enter a hospital for general training?—*A Seven Years' Reader.*

You have passed the limit for the larger schools. But you might hear of a matron of one of the cottage hospitals who would train you, if you advertise.

*Change of Name.*

(24) Would you kindly tell me if it is necessary to inform the Midwives' Institute that, since I took the L.O.S., I have married? Also if, now that the Midwives Bill has passed, I am supposed to have my name registered?—*G. B.*

Of course, if you are a member, you must write and inform the institute of your change of name. The Secretary will also advise you as to the steps necessary to register yourself as midwife.

**Important Nursing Textbooks.**

"The Nursing Profession: How and where to Train." 2s. net; 2s. 4d. post free.

"A Handbook for Nurses." (New Edition). 5s. net; 5s. 4d. post free.

"Practical Guide to Surgical Bandaging and Dressings." By Wm. Johnson Smith, F.R.C.S. 2s. post free.

"The Nurses' Dictionary of Medical Terms and Nursing Treatment." By Honnor Morten. 2s. post free.

"The Human Body: its Personal Hygiene and Practical Physiology." By B. P. Colton. 5s. post free.

"Art of Feeding the Invalid." (Popular Edition). 1s. 6d. post free.

"On Preparation for Operation in Private Houses. By Stanmore Bishop, F.R.C.S. 6d. post free.

